# Supplementary material for: A DNA demethylase reduces seed size by decreasing the DNA methylation of AT-rich transposable elements in soybean
Source: Commun Biol. 2024 May 21;7:613. doi: 10.1038/s42003-024-06306-2 (PMC11109123; doi:10.1038/s42003-024-06306-2)
Supplement: Supplementary file 2 — Supplementary Information [file 42003_2024_6306_MOESM2_ESM.pdf]

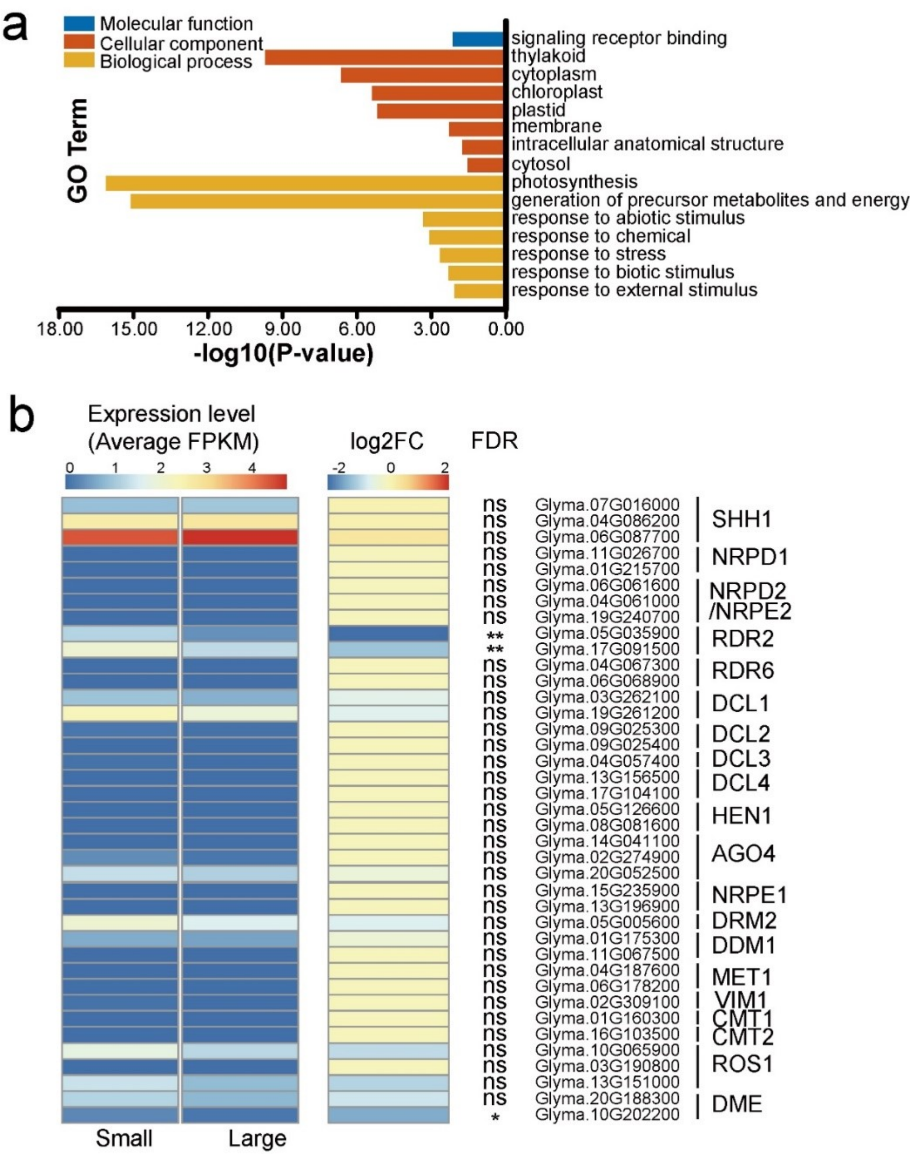

Supplementary Fig. 1 Gene expression of factors involved in DNA methylation in the small- and large-seed groups.

a. GO enrichment of upregulated DEGs between the two groups, with yellow bars highlighting molecular functions and blue bars depicting biological processes. The -log of the enrichment p value was used to visualize the significance of differential gene expression, with longer bars representing greater enrichment.

b. Heatmap of the expression levels (FPKM) of epigenetic genes in the small- and large-seed groups. Statistical disparities between means were evaluated using unpaired one-tailed t-tests, signifying significance with *FDR < 0.05 (significant), ** FDR < 0.01 (highly significant), ns FDR > 0.05 (not significant).

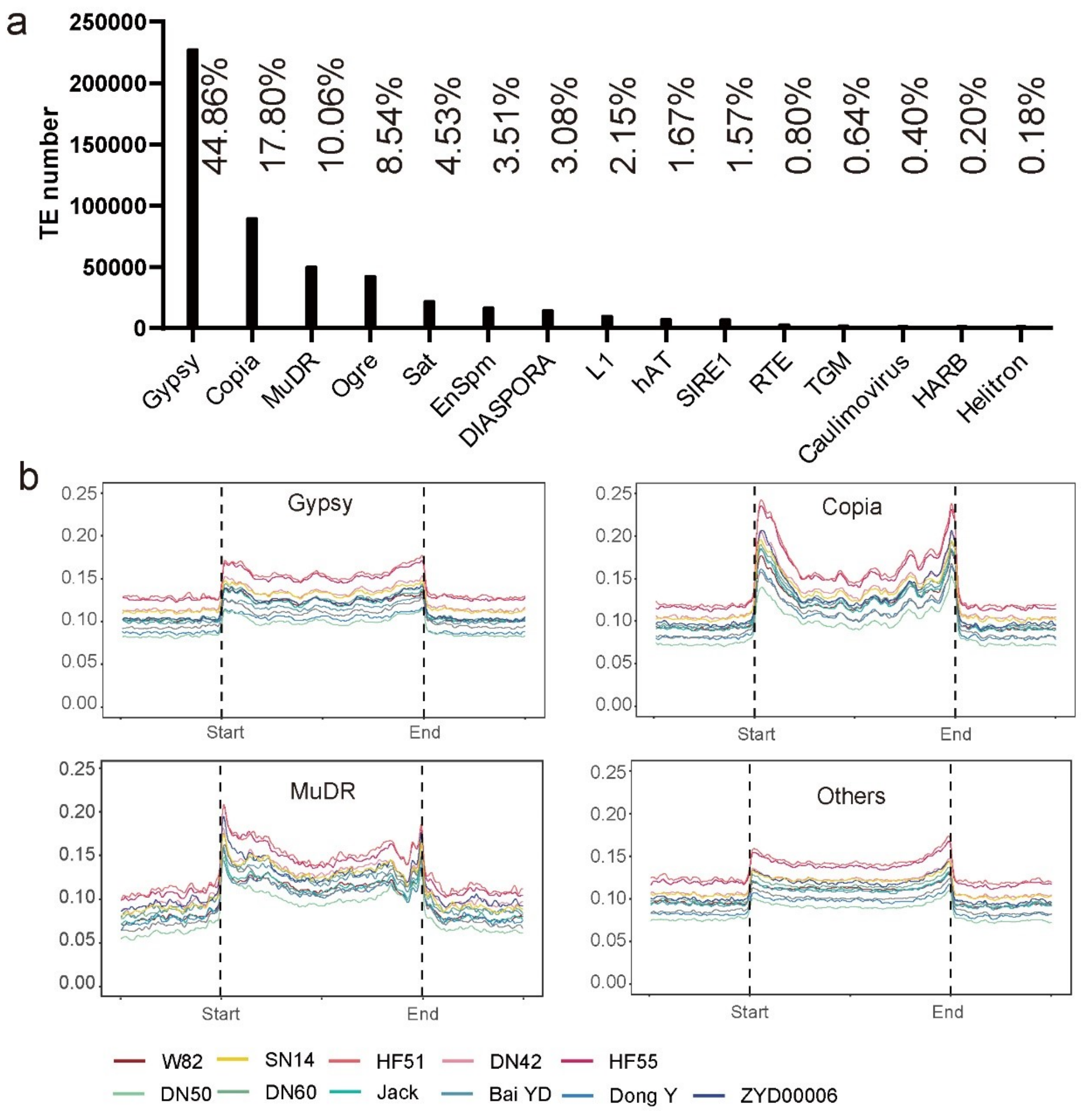

Supplementary Fig. 2 DNA methylation levels in different types of TEs in the eleven germplasms.

a. Percentages of different types of TEs in the genome of soybean.

b. The CHH methylation levels of the four TEs in the eleven soybean ecotypes are depicted in a line graph. The X-axis indicates the TE body (start to end) and 2 kb flanking regions.

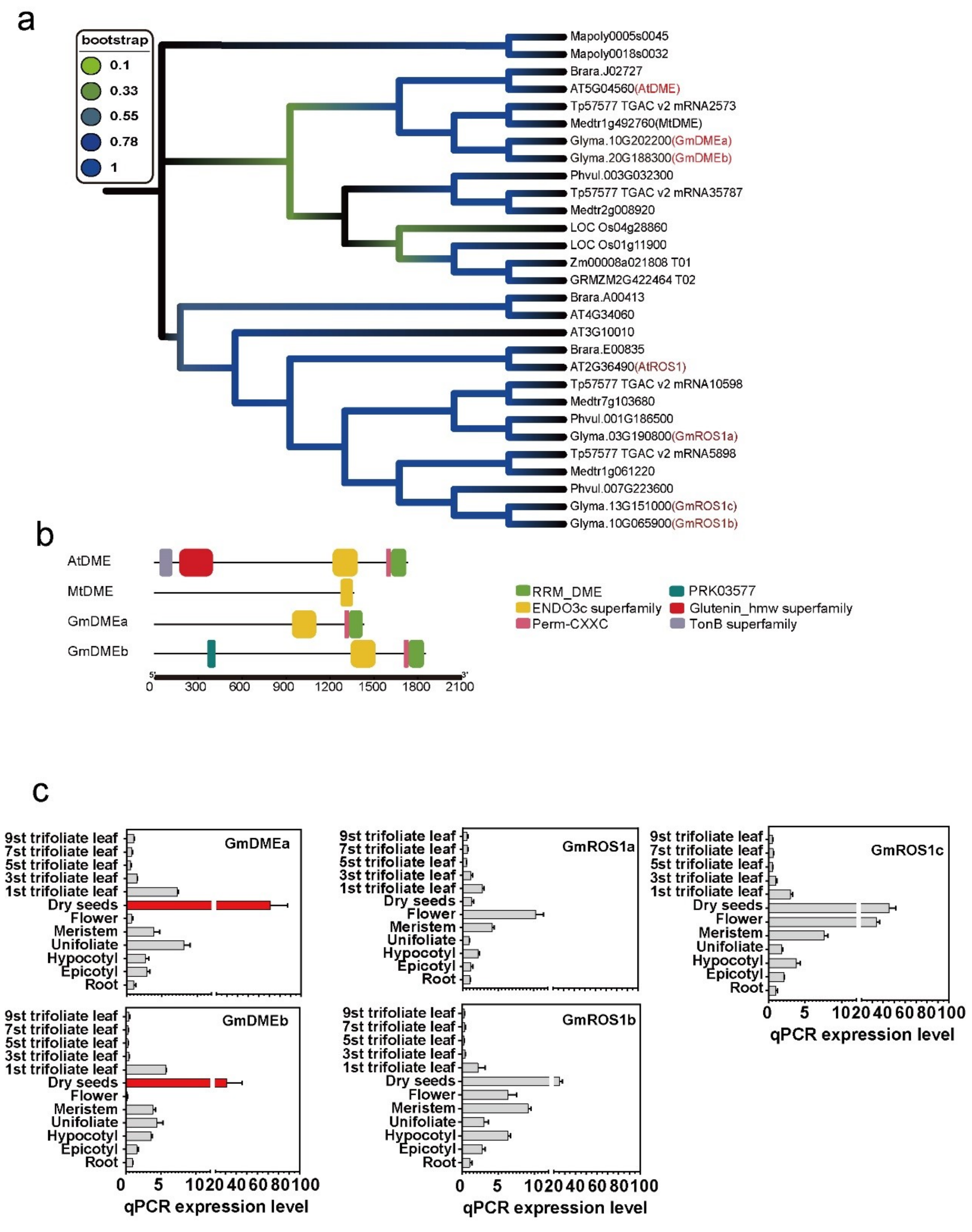

Supplementary Fig. 3 Proximity analysis, conserved domains, and expression pattern of demethylases.

a. Phylogenetic tree constructed with protein sequences of DME family proteins from soybean (*Glycine max*), alfalfa (*Medicago truncatula*), kidney bean (*Phaseolus vulgaris*), red clover (*Trifolium pratense*), *Arabidopsis thaliana*, potato (*Solanum tuberosum*), tomato (*Solanum lycopersicum*), maize (*Zea mays*), rice (*Oryza sativa*) and *Marchantia polymorpha*. The DME

protein from *Marchantia polymorpha* was used as an outgroup. Tree computed via maximum likelihood using MEGA 10.0 with 1000 bootstrap replicates (see Methods section for details).

b. Protein domains of DME are conserved in GmDMEa (Glyma.10G202200), GmDMEb (Glyma.20G188300), MtrDME (Medtr.1g492760) and AtDME (At5G04560). Conserved domains were identified using the NCBI conserved domain search tool against the CDD v3.20 database. Domain details are listed in the table. Light green box: RRM_ DME; yellow box: ENDO3C superfamily; pink box: Perm CXXC; dark green box: PRK03577; red box: Glutenin_ hmw superfamily; grey box: TonB superfamily.

c. Transcript levels of *GmDMEa*, *GmDMEb*, *GmROS1a* (*Glyma.03g190800*), *GmROS1b* (*Glyma.10g065900*) and *GmROS1c* (*Glyma.13g151000*) in specific tissues determined by qRT–PCR. RNA samples were extracted from the root, epicotyl, hypocotyl, unifoliate leaf, shoot meristem, flower, dry seeds, 1st trifoliate leaf, 3rd trifoliate leaf, 5th trifoliate leaf, 7th trifoliate leaf, and 9th trifoliate leaf of DN50 plants.

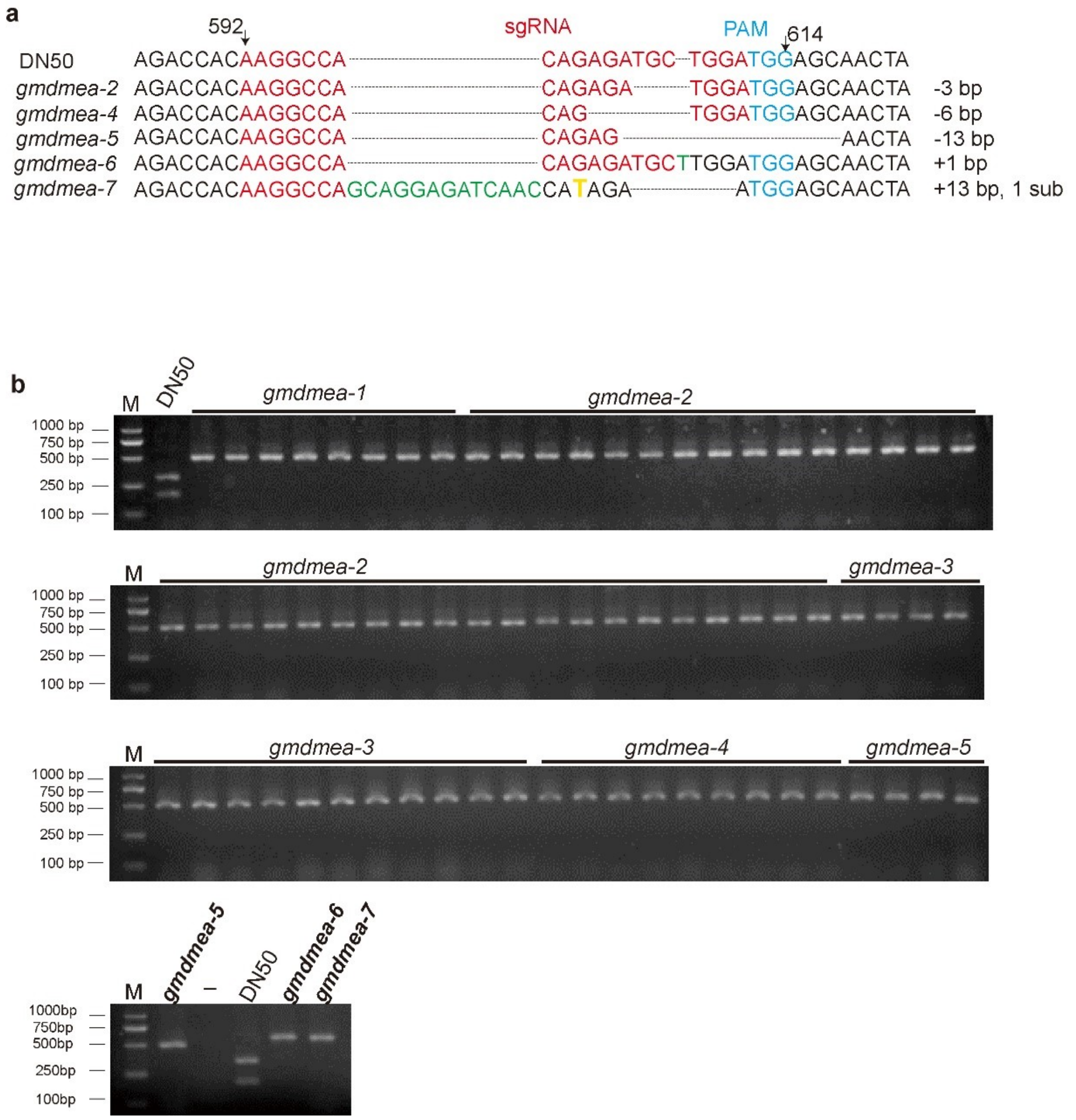

Supplementary Fig. 4 Genotyping of different lines of *gmdme* mutants

a Mutant sequences of *gmdmea-2*, *gmdmea-4*, *gmdmea-5*, *gmdmea-6*, and *gmdmea-7*. The sgRNA sequence (red) and the protospacer adjacent motif (PAM, blue) sequence shown in DN50. The numbers (592 and 614) above the nucleotide sequences are relative to the start codon of *GmDMEa*. The dashed lines indicate the deletion in the mutants. The numbers (-3, -6, -13, +1, +13) at the end of the sequence are deleted/insertion nucleotide numbers, and 1 sub means 1 substitute nucleotide.

b CAPS markers were used to identify the mutations in the *gmdmea* mutants. PCR products amplified by specific primers from DN50 (WT) and the *gmdmea-1*, *gmdmea-2*, *gmdmea-3*, *gmdmea-4*, and *gmdmea-5* plants were digested by *SfaN I* (GCATCN5/9), and *gmdmea-6* and *gmdmea-7* plants were digested by *Xcm I* (CCANNNNNNNNNTGG). The primers used in this experiment are listed in Table 12. M represents the DNA ladder.

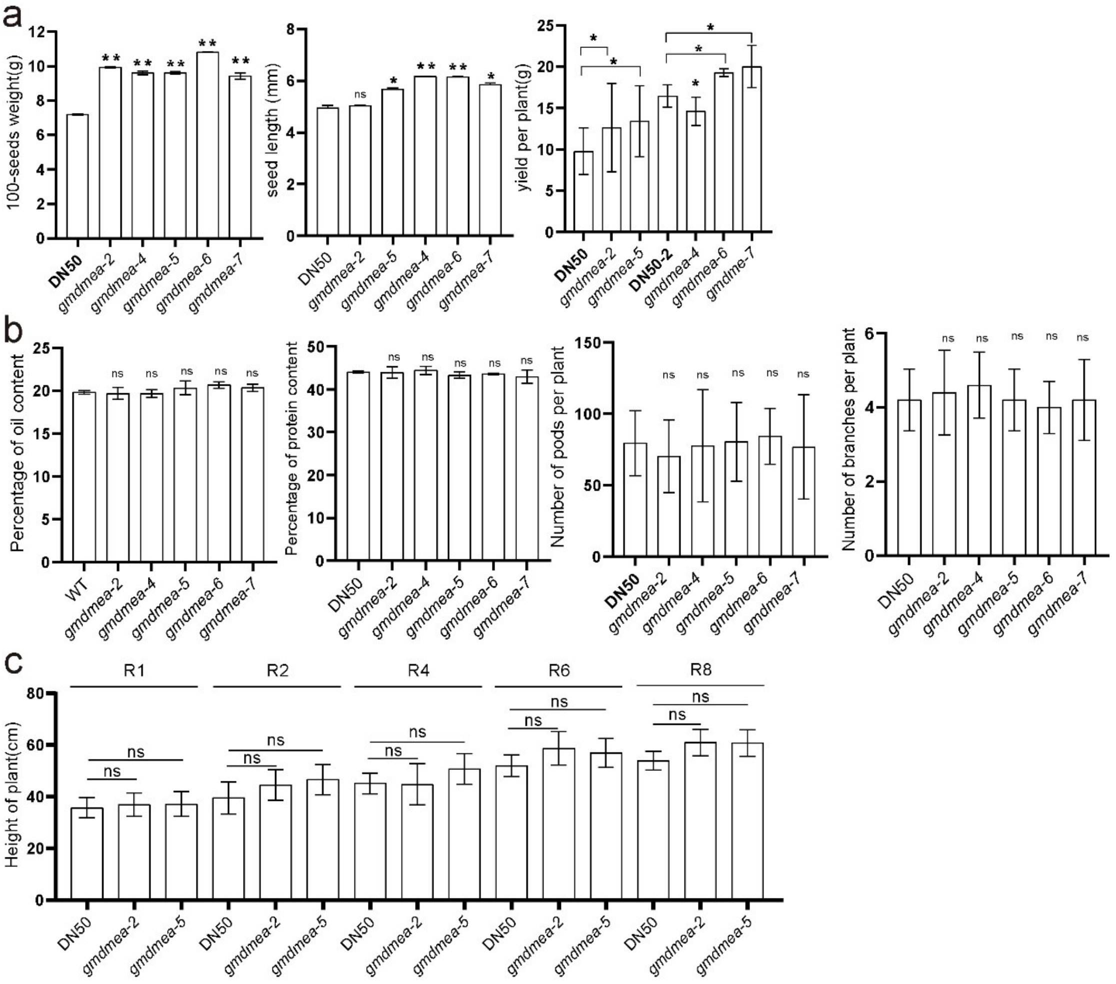

Supplementary Fig. 5 Phenotype of *gmdmea* mutants.

a. The 100-seed weight, seed length and yield of DN50, *gmdmea-2*, *gmdmea-4*, *gmdmea-5*, *gmdmea-6*, and *gmdmea-7*. Values are means ± SDs (n = 10).

b. Percentage of oil content and protein content, number of branches and pods of DN50, *gmdmea-2*, *gmdmea-4*, *gmdmea-5*, *gmdmea-6*, *gmdmea-7* plants in the T2 generation. Values are means ± SDs (n = 6). The number of pods and branches per plant of all DN50, *gmdmea-1* and *gmdmea-3* plants in the T2 generation were counted. Values are means ± SDs (n ≥ 9).

c. Height of plants in different growth stages from R1 to R8 and the harvestable stage. R, reproductive stage. From the R1 stage to R8, pods achieve mature colouration, which signals physiological maturity, and are finally harvestable. Values are means ± SDs (n ≥ 9). Significant differences in mean values relative to the mean value of wild-type plants are indicated by unpaired two-tailed Student's t tests (*, p < 0.05, **, p < 0.01, ns, p > 0.05).

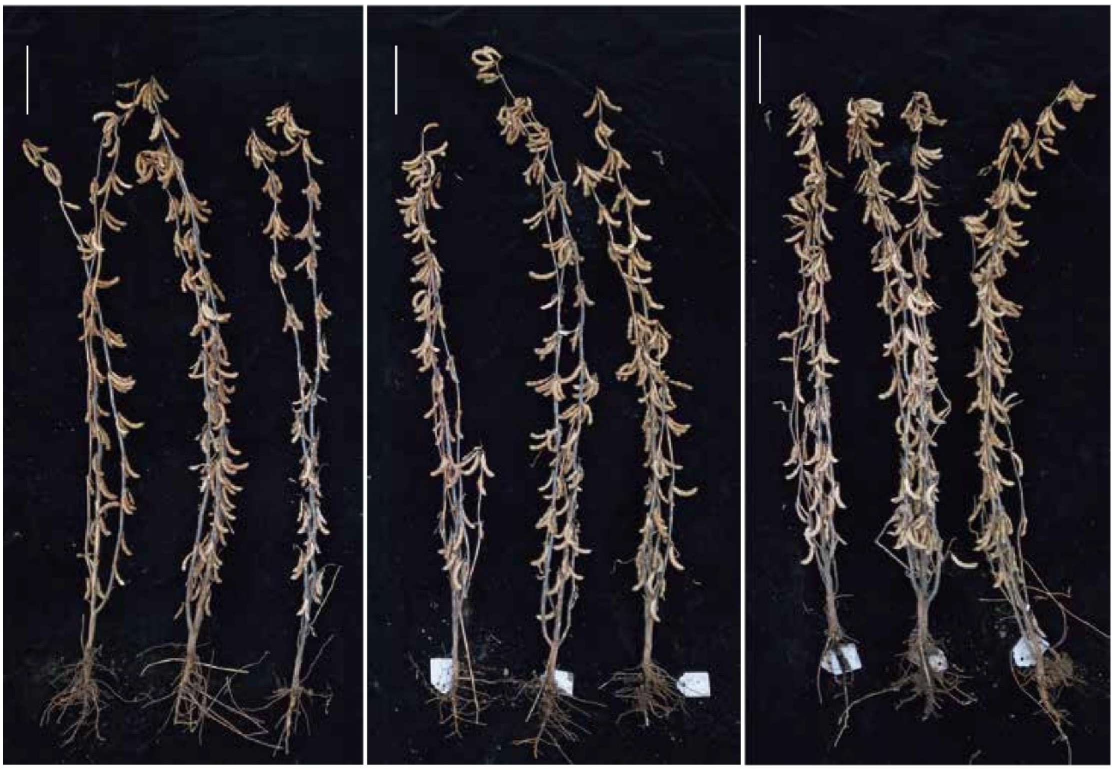

DN50 *gmdmea-1* *gmdmea-3*

Supplementary Fig. 6 Whole harvested plants of DN50 and *gmdmea* mutants. Scale bar = 10 cm.

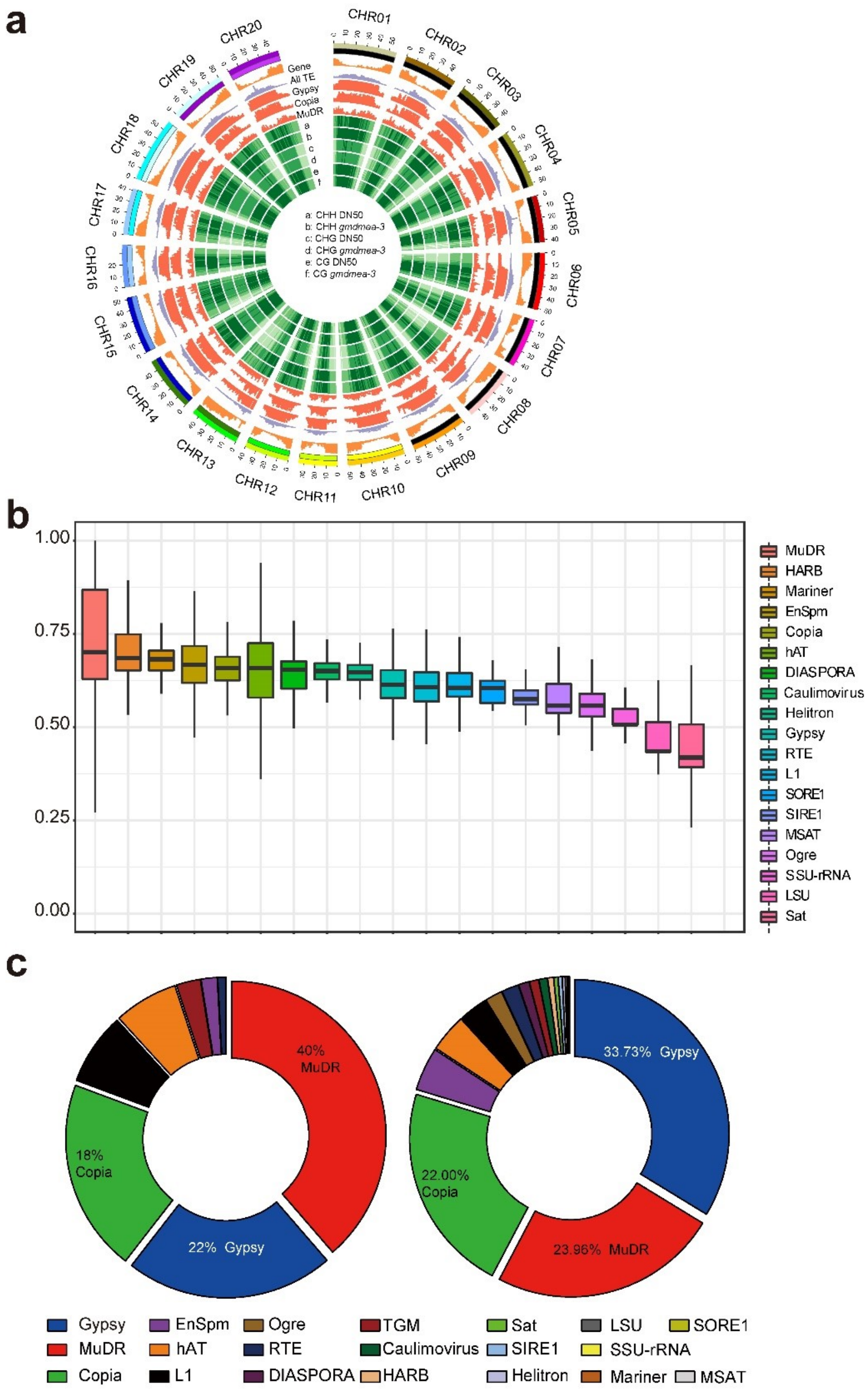

Supplementary Fig. 7 Characteristics of TEs in soybean.

a. Circos plot indicating the genome-wide distribution of CG, CHG and CHH methylation in wild type (DN50) and the mutant (*gmdmea-3*).

b. AT ratio of different TE types in the soybean whole genome

c. Distribution of TEs located upstream of DME-regulated DEGs (left) and all genes (right) in the whole soybean genome. Extend fig 4h.

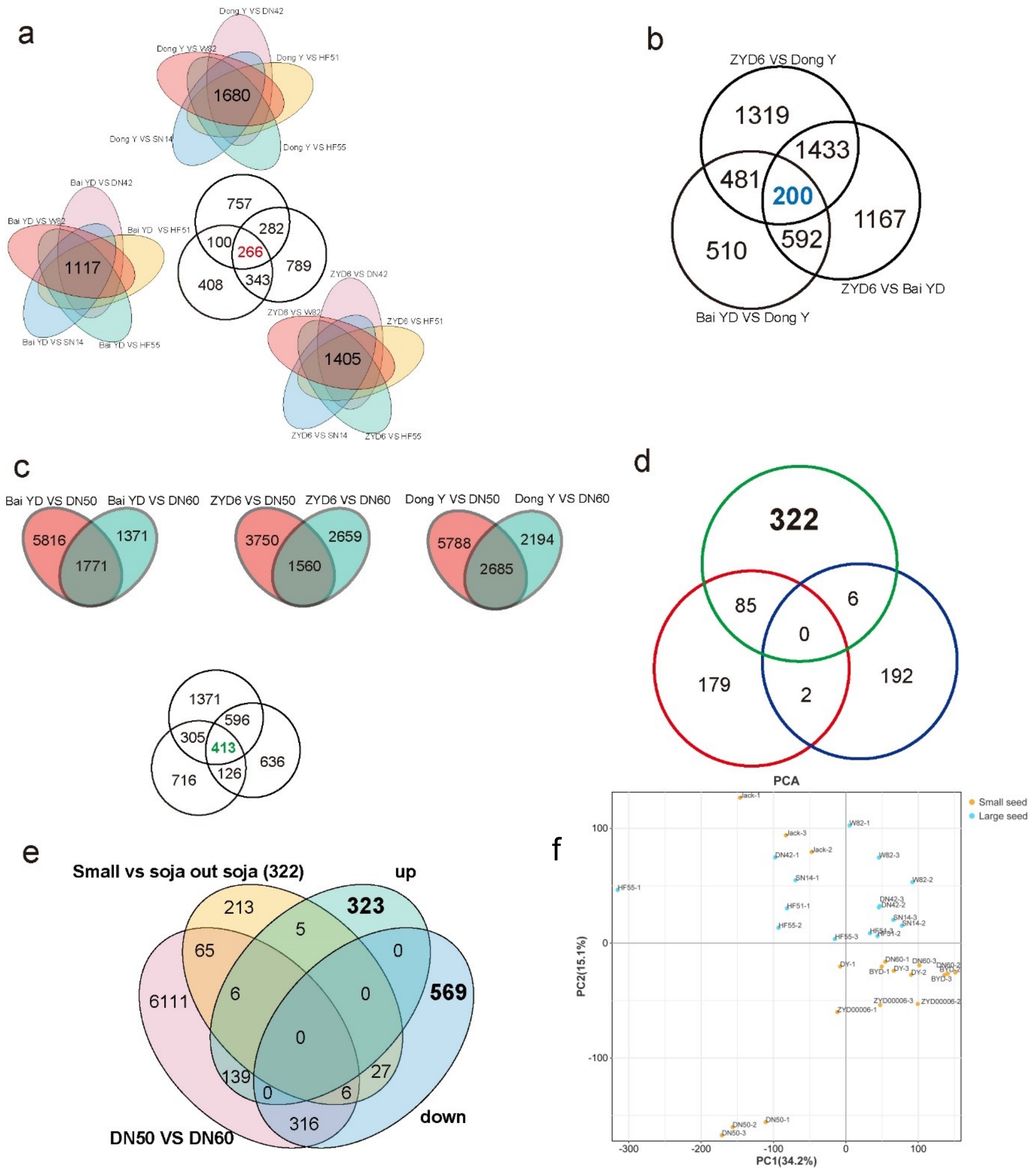

Supplementary Fig. 8 Genes that only regulate soybean seed size.

a. Venn diagrams illustrate the comparisons between wild soybeans and large-seed cultivars, revealing a total of 266 differentially expressed genes (DEGs).

b. Venn diagrams illustrate the comparisons between 3 wild soybeans, revealing a total of 200 differentially expressed genes (DEGs).

c. Venn diagrams illustrate the comparisons between wild beans and small seed cultivars, revealing a total of 413 differentially expressed genes (DEGs).

d. Venn diagrams illustrate the DEGs between small grains and wild beans, and the effect of wild

beans themselves was excluded.

e. The Venn diagram depicts the differentially expressed genes (DEGs) between DN50 and *gmdmea-3*. After excluding the DEGs between DN50-DN60 and wild-small seeds, 569 genes remained that were closely associated with seed size.

f. Principal component analysis (PCA) of RNA-seq data distinguishes between large-seed and small-seed soybean germplasm groups

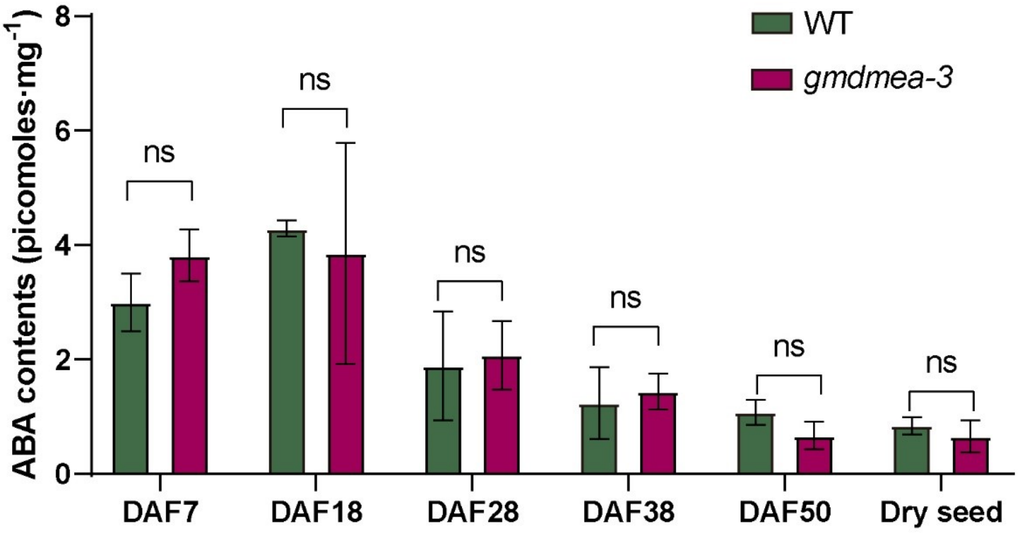

Supplementary Fig. 9 Determination of endogenous ABA in seeds.

Endogenous ABA content of seeds at different developmental stages of wild type and mutant plants. Displayed are mean values ± SD, with a minimum of two biological replicates. Statistical disparities between means were evaluated using unpaired two-tailed t-tests, signifying significance with ns $p > 0.05$ (not significant).

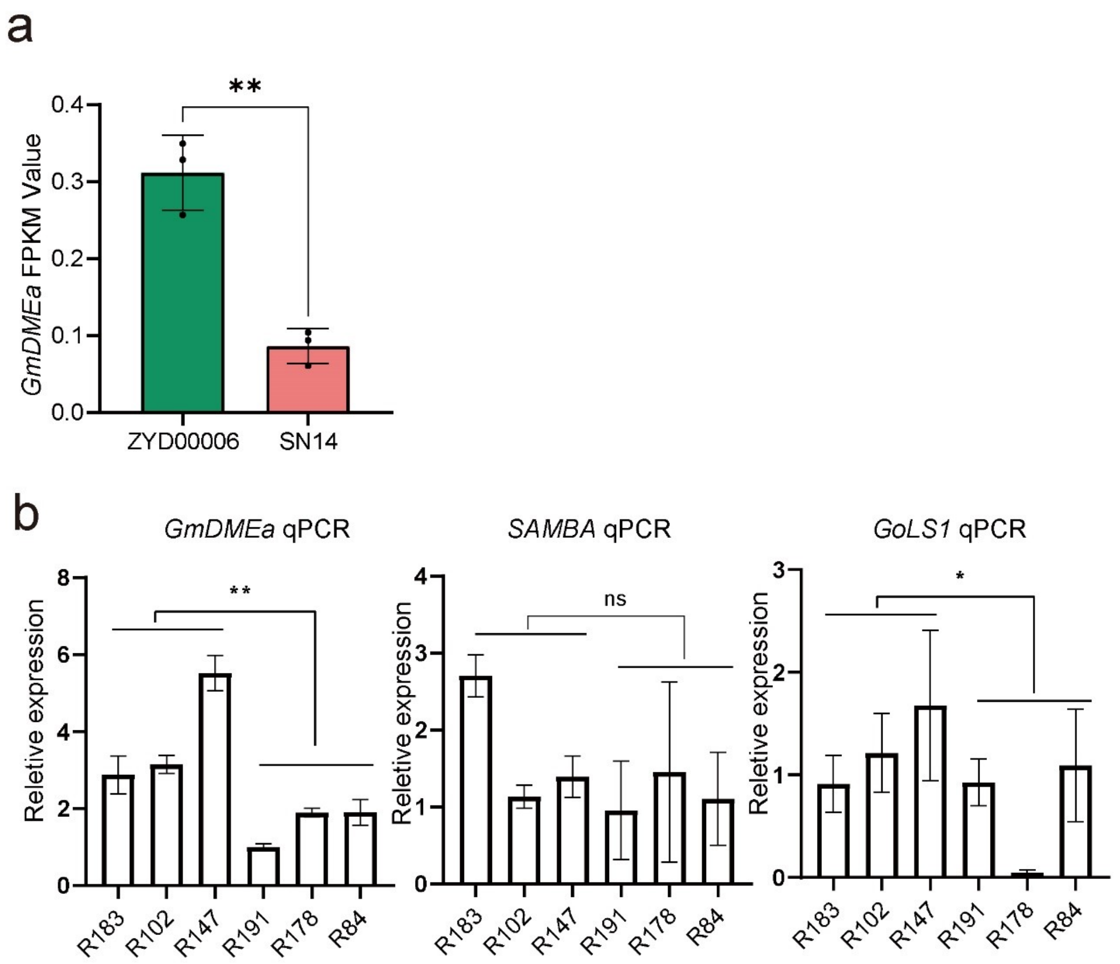

Supplementary Fig. 10 Expression of *GmDMEa*, *SAMBA*, and *GoLS1* in CSSL offspring

a. FPKM values show the expression level of *GmDMEa*. Data are shown as the mean ± SD (n = 3).

b. RT-PCR was used to measure the expression levels of *GmDMEa*, *SAMBA*, and *GoLS1*. Data are shown as the mean ± SD (n = 9).

Statistical assessments were performed using unpaired two-tailed t-tests with equal variance. Significance is indicated as follows: *$p < 0.05$ (significant), **$p < 0.01$ (highly significant), ns $p > 0.05$ (not significant).

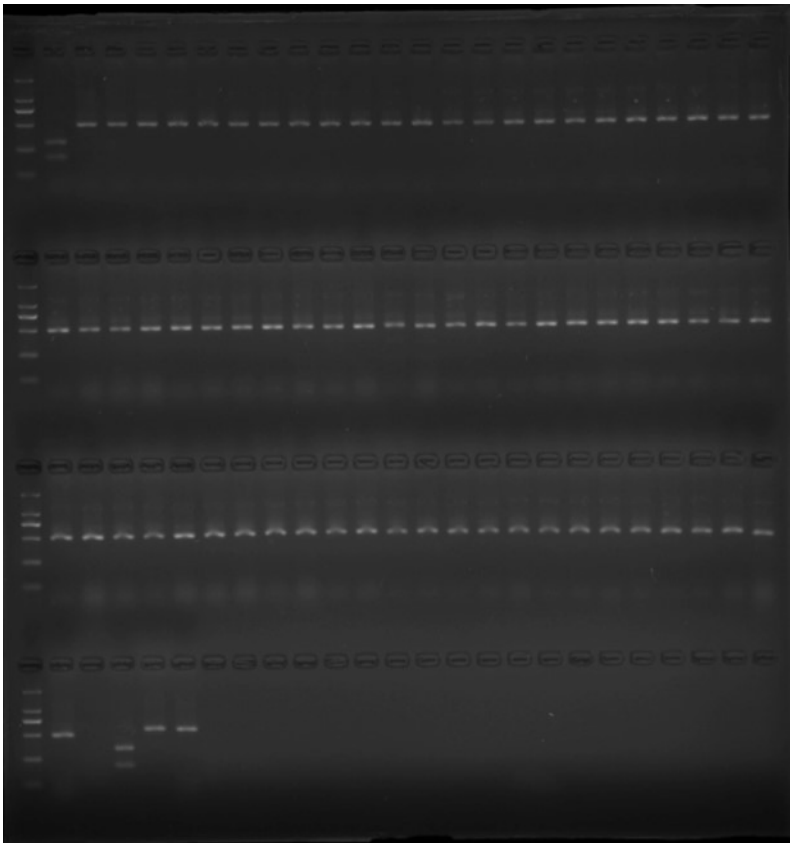

Supplementary Fig. 11 Original Gel Electrophoresis Results of CAPS Experiment
