# Supplementary material for: A DNA demethylase reduces seed size by decreasing the DNA methylation of AT-rich transposable elements in soybean
Source: Commun Biol. 2024 May 21;7:613. doi: 10.1038/s42003-024-06306-2 (PMC11109123; doi:10.1038/s42003-024-06306-2)
Supplement: Supplementary file 3 — Description of Additional Supplementary Files. [file 42003_2024_6306_MOESM3_ESM.docx]

Description of Additional Supplementary Files

**File name**: Supplementary Data 1

**Description**: Differentially expressed genes (DEGs) in the small and large seed group.

**File name**: Supplementary Data 2

**Description**: GO categories of DEGs in the small and large seed group.

**File name**: Supplementary Data 3

**Description**: Differentially Methylated Region (DMRs) in the small and large seed group.

**File name**: Supplementary Data 4

**Description**: Comparison of functional domains.

**File name**: Supplementary Data 5

**Description**: Homology analysis of DMEs.

**File name**: Supplementary Data 6

**Description**: List of the Seven *gmdmea* mutations.

**File name**: Supplementary Data 7

**Description**: DMRs between DN50 and *gmdmea-3*

**File name**: Supplementary Data 8

**Description**: DEGs between DN50 and *gmdmea-3*.

**File name**: Supplementary Data 9

**Description**: 49 CHH-hyper-DEG.

**File name**: Supplementary Data 10

**Description**: DEGs closely related to seed size in soybean.

**File name**: Supplementary Data 11

**Description**: GO categories of DEGs closely related to seed size in soybean.

**File name**: Supplementary Data 12

**Description**: Summary of seed methylomes from wild type (Dongnong50), mutant (*gmdmea-3*) and other soybean germplasms, related to Figure 2.

**File name**: Supplementary Data 13

**Description**: Summary of seed transcriptome from wild type (Dongnong50), mutant (*gmdmea-3*) and other soybean germplasms.

**File name**: Supplementary Data 14

**Description**: All primers used in this study.
