# Supplementary material for: A DNA demethylase reduces seed size by decreasing the DNA methylation of AT-rich transposable elements in soybean
Source: Commun Biol. 2024 May 21;7:613. doi: 10.1038/s42003-024-06306-2 (PMC11109123; doi:10.1038/s42003-024-06306-2)
Supplement: Supplementary file 18 — reporting-summary [file 42003_2024_6306_MOESM18_ESM.pdf]

nature portfolio

Corresponding author(s): Linan Xie

Last updated by author(s): Mar 22, 2024

Reporting Summary

Nature Portfolio wishes to improve the reproducibility of the work that we publish. This form provides structure for consistency and transparency in reporting. For further information on Nature Portfolio policies, see our Editorial Policies and the Editorial Policy Checklist.

Statistics

For all statistical analyses, confirm that the following items are present in the figure legend, table legend, main text, or Methods section.

n/a	Confirmed	
☐	☒	The exact sample size (*n*) for each experimental group/condition, given as a discrete number and unit of measurement
☐	☒	A statement on whether measurements were taken from distinct samples or whether the same sample was measured repeatedly
☐	☒	The statistical test(s) used AND whether they are one- or two-sided<br>*Only common tests should be described solely by name; describe more complex techniques in the Methods section.*
☐	☒	A description of all covariates tested
☐	☒	A description of any assumptions or corrections, such as tests of normality and adjustment for multiple comparisons
☐	☒	A full description of the statistical parameters including central tendency (e.g. means) or other basic estimates (e.g. regression coefficient) AND variation (e.g. standard deviation) or associated estimates of uncertainty (e.g. confidence intervals)
☐	☒	For null hypothesis testing, the test statistic (e.g. *F*, *t*, *r*) with confidence intervals, effect sizes, degrees of freedom and *P* value noted<br>*Give P values as exact values whenever suitable.*
☒	☐	For Bayesian analysis, information on the choice of priors and Markov chain Monte Carlo settings
☒	☐	For hierarchical and complex designs, identification of the appropriate level for tests and full reporting of outcomes
☐	☒	Estimates of effect sizes (e.g. Cohen's *d*, Pearson's *r*), indicating how they were calculated

Our web collection on statistics for biologists contains articles on many of the points above.

Software and code

Policy information about availability of computer code

Data collection	Data was obtained after sequencing (bi-sulphite and mRNA) of the genomic DNA and total RNA samples.
Data analysis	Only open source software were used. All softwares used are listed in the Methods section.<br>For example,MEGA 10.0 for Phylogenetic tree; WGBS data were processed by FastQC, BSmap; RNAseq data were processed by Fastp, Hisat2, SAMtools, StringTie. Differentially expressed genes using DEseq2 package in R.

For manuscripts utilizing custom algorithms or software that are central to the research but not yet described in published literature, software must be made available to editors and reviewers. We strongly encourage code deposition in a community repository (e.g. GitHub). See the Nature Portfolio guidelines for submitting code & software for further information.

Data

Policy information about availability of data

All manuscripts must include a data availability statement. This statement should provide the following information, where applicable:

- Accession codes, unique identifiers, or web links for publicly available datasets
- A description of any restrictions on data availability
- For clinical datasets or third party data, please ensure that the statement adheres to our policy

The datasets presented in this study can be found in online repositories. The names of the repository/repositories and accession number(s) can be found below: National Center for Biotechnology Information (NCBI) Sequence Read Archive PRJNA954182 and PRJNA954174.

Research involving human participants, their data, or biological material

Policy information about studies with human participants or human data. See also policy information about sex, gender (identity/presentation), and sexual orientation and race, ethnicity and racism.

Reporting on sex and gender	N/A
Reporting on race, ethnicity, or other socially relevant groupings	N/A
Population characteristics	N/A
Recruitment	N/A
Ethics oversight	N/A

Note that full information on the approval of the study protocol must also be provided in the manuscript.

Field-specific reporting

Please select the one below that is the best fit for your research. If you are not sure, read the appropriate sections before making your selection.

☒ Life sciences ☐ Behavioural & social sciences ☐ Ecological, evolutionary & environmental sciences

For a reference copy of the document with all sections, see nature.com/documents/nr-reporting-summary-flat.pdf

Life sciences study design

All studies must disclose on these points even when the disclosure is negative.

Sample size	Total 33 RNAseq and 11 WGBS of three types of wild soybean (G. soja) ,one mutant(gmdmea-3) and eight cultivars. Each transcriptome sample consists of three biological replicates and one biological replicate for genome-wide methylation samples
Data exclusions	No data was excluded
Replication	see Sample size fo Γreplication information
Randomization	No randomization
Blinding	No blinding

Reporting for specific materials, systems and methods

We require information from authors about some types of materials, experimental systems and methods used in many studies. Here, indicate whether each material, system or method listed is relevant to your study. If you are not sure if a list item applies to your research, read the appropriate section before selecting a response.

Materials & experimental systems

n/a	Involved in the study	
☒	☐	Antibodies
☒	☐	Eukaryotic cell lines
☒	☐	Palaeontology and archaeology
☒	☐	Animals and other organisms
☒	☐	Clinical data
☒	☐	Dual use research of concern
☐	☒	Plants

Methods

n/a	Involved in the study	
☒	☐	ChIP-seq
☒	☐	Flow cytometry
☒	☐	MRI-based neuroimaging

Plants

Seed stocks	Three types of wild soybean (G. soja) and eight cultivars, including ZYD00006 (ZYD6), Baiyangdian (Bai YD) and Dongying (Dong Y); Williams 82 (W82), Jack, Dongnong 50 (DN50), Dongnong 60 (DN60), Dongnong 42 (DN42), Hefeng 51 (HF51), Hefeng 55 (HF55) and Suinong 14 (SN14), were used in this study. All soybean materials used in this study were obtained from the Chinese Academy of Agricultural Sciences (CAS), Northeast Forestry University (NEFU), and Northeast Agricultural University (NEAU). Six progeny populations, R183, R147, R102, R191, R178 and R84 of CSSL derived from SN14 × ZYD6, were obtained from Northeast Agricultural University. All gmdmea mutants seeds were stored in Northeast Forestry University
Novel plant genotypes	For information on all gene editing mutants, see the "Methods" section of the manuscript.
Authentication	For information on all gene editing mutants, see the manuscript and Table 6.
